# *Flos lonicerae* and *Baikal skullcap* Extracts Improved Laying Performance of Aged Hens Partly by Modulating Antioxidant Capacity, Immune Function, Cecal Microbiota and Ovarian Metabolites

**DOI:** 10.3390/ani15192882

**Published:** 2025-10-01

**Authors:** Xu Yu, Jun Li, Ruomu Peng, Xiaodong Zhang, Wanfu Yue, Yufang Wang, Yahua Lan, Yongxia Wang

**Affiliations:** 1 Key Laboratory of Applied Technology on Green-Eco-Healthy Animal Husbandry of Zhejiang Province, College of Animal Science and Technology/College of Veterinary Medicine, Zhejiang A&F University, Hangzhou 311300, China; 13456636461@163.com (X.Y.); 17355558106@163.com (J.L.); lovezuole@163.com (R.P.); 20120058@zafu.edu.cn (X.Z.); 20110058@zafu.edu.cn (W.Y.); 2 Qujiang District Animal Husbandry and Veterinary Station, Quzhou 324000, China; wang258002025@163.com (Y.W.); lyh371391@zuaa.zju.edu.cn (Y.L.)

**Keywords:** aged laying hens, *Flos lonicerae* and *Baikal skullcap* extracts, follicular development, cecal microbiota, ovarian metabolomic

## Abstract

**Simple Summary:**

During the late laying period, reduced egg production causes economic losses, while the industry aims to extend laying cycles, emphasizing the need to improve the laying performance of aged hens. The decline in laying rate is mainly associated with diminished ovarian function. Nutritional strategies that support ovarian function are therefore crucial for aged hens. *Flos lonicerae* and *Baikal skullcap* Extracts (PE) have been reported to enhance laying rate of laying hens. Thus, we speculate that dietary supplemented with PE may improve ovarian function of older hens. In this study, PE supplementation improved laying performance, immunity, antioxidant capacity, and ovarian function of aged hens. Specifically, PE supplementation increased pre-hierarchical follicle numbers, ovarian index, and serum estrogen levels. 16S rRNA sequencing revealed that PE induced alterations in the composition of the cecal microbiota, with these changes primarily linked to the production of short-chain fatty acids. Ovarian metabolomics further indicated that PE regulated metabolites related to follicle growth, estrogen secretion, and antioxidant and anti-inflammatory responses. In summary, PE improved laying performance by enhancing antioxidant and immune functions, promoting follicular development and estrogen secretion, and modulating gut microbiota and ovarian metabolites. These findings offer a mechanistic foundation for the nutritional regulation of ovarian function in aged hens.

**Abstract:**

The aim of this study was to evaluate the effects of *Flos lonicerae* and *Baikal skullcap* extracts (PE) on laying performance, antioxidant capacity, immune function, follicular development, estrogen secretion, ovarian metabolomics, and cecal microbiota in aged laying hens. The total number of 70-week-old XinYang Black-Feathered laying hens was 240. These hens were randomly divided into two groups, with each group consisting of six replicates of 20 birds. Control (CON) group was fed a basal diet, whereas the PE group received the same basal diet supplemented with 500 mg/kg of PE. The duration of the experiment was 10 weeks. The findings indicated that the supplementation of PE improved laying performance, antioxidant capacity, and immune function. This was reflected by significant increases (*p* < 0.05) in laying rate, feed conversion ratio, antioxidant indicators (such as glutathione peroxidase, total antioxidant capacity, and catalase), and immunoglobulin levels. Additionally, there were notable decreases (*p* < 0.05) in the malondialdehyde levels and pro-inflammatory markers. Moreover, the PE group exhibited a greater number of large yellow and white follicles, as well as higher serum estrogen levels, compared to the CON group (*p* < 0.05). 16S rRNA sequencing revealed that PE supplementation altered the composition of the cecal microbiota by increasing *Ruminococcus_torques_group*, *Butyricoccus* and *Christensenellaceae_R-7_group* abundances and decreasing *Bacteroides*, *Prevotellaceae_UCG-001* and *Megamonas* abundances (at genus level), which are primarily associated with short-chain fatty acid production. Ovarian metabolomic analysis showed that the major metabolites altered by PE supplementation were mainly involved in follicular development, estrogen biosynthesis, anti-inflammatory and antioxidant properties. Moreover, changes in both the cecal microbiota (at genus level) and ovarian metabolites were strongly correlated with laying performance, antioxidant status, and immune function. In conclusion, PE supplementation improved laying performance in aged hens by enhancing antioxidant, immune, and ovarian functions, promoting follicular development and estrogen secretion, and modulating the gut microbiota and ovarian metabolites. These findings will offer novel insights into the mechanisms that underlie egg production in the ovaries of aged poultry.

## 1. Introduction

During the late laying period, the continuous decline in laying rate leads to economic losses for poultry farms [1,2]. On the other hand, in recent years, the egg industry has been actively exploring strategies to extend the laying cycle in order to maximize the utilization of housing facilities and equipment [3]. Thus, it is essential to find effective ways to improve the laying performance of aged hens.

The decline in laying rate in aged hens is mainly linked to ovarian oxidative stress [4], which in turn accelerates ovarian aging. This results in a diminished number of follicles and a decrease in estrogen levels, which consequently leads to a reduced laying rate in older hens [1]. Therefore, identifying appropriate nutritional strategies to improve ovarian function in aged laying hens is of great importance.

*Flos lonicerae*, cultivated in China for thousands of years. Its various products, including herbal drinks, wine, tea, and traditional Chinese medicine preparations such as honeysuckle granules, are highly valued [5,6]. China is the primary producer of *Baikal skullcap* for medicinal purposes, and its extract is widely applied in diverse industries, including pharmaceuticals, nutraceuticals, cosmetics, and food and beverages. Both plants hold significant medicinal, economic, and ecological value [7]. Chlorogenic acid (CGA) and baicalin are the major active components of *Flos lonicerae* and *Baikal skullcap*, respectively [8]. Both baicalin and CGA demonstrate a range of biological activities, encompassing antioxidant, anti-inflammatory, antibacterial, and antiviral effects [9]. A study by Xie et al. [10] showed that supplementing the diet of 52-week-old Lohman pink-shell hens with 0.1% *Flos lonicerae* and 0.1% *Baikal skullcap* extracts for 12 weeks significantly decreased plasma malondialdehyde levels and increased hen-day egg production. Additionally, Liu et al. [11] demonstrated that dietary supplementation with 300, 500, or 1000 mg/kg *Flos lonicerae* extract for 11 weeks significantly improved hen-day egg production in 83-week-old Roman Pink laying hens.

Therefore, we hypothesized that supplementation with *Flos lonicerae* and *Baikal skullcap extracts* (PE) could improve laying performance of aged hens by enhancing ovarian function. However, a comprehensive and in-depth understanding of how PE precisely regulates ovarian function is still lacking, particularly with regard to the key metabolites, metabolic pathways and gut microbiota through which this regulatory process is mediated. Consequently, a feeding trial was undertaken to evaluate the effect of PE supplementation on laying performance, antioxidant capacity, immune function, follicular development, estrogen secretion, ovarian metabolomics, and cecal microbiota of aged laying hens. The aim of this study is to identify key metabolites targeting the enhancement of ovarian function, thereby providing a theoretical basis for their application as novel additives in reproductive health regulation, and offering scientific support for the development of personalized nutritional interventions based on core ovarian metabolites.

## 2. Materials and Methods

### 2.1. Experimental Design, Birds and Diets

The experimental procedures were conducted in adherence to the Chinese Guidelines for Animal Welfare and were approved by the Animal Care and Use Committee at Zhejiang A&F University.

A total of 240 healthy, 70-week-old XinYang Black-Feathered laying hens with a comparable laying rate of 71% were randomized into two groups of six replicates, each consisting of 20 birds. Initial body weight and laying rate were standardized across all replicates after a two-week pre-test period. In the pre-test phase, all birds were given a basal diet consisting of corn-soybean meal, as outlined in Table A1. The composition of this diet met the nutritional requirements for laying hens as per the NY/T-33-2004 feeding standard (China National Standard, 2004) [12].

The two experimental groups were as follows: the control (CON) group, where the birds were fed the same corn-soybean meal basal diet as during the pre-test; and the PE group, where the birds received the basal diet supplemented with 500 mg/kg of PE. This optimal dose of PE was selected based on prior findings from Liu et al. [11]. The PE consisted of *Flos lonicerae* extract (10% chlorogenic acid) and *Baikal skullcap* extract (90% baicalin) in a 3:2 ratio, providing final concentrations of 300 mg/kg chlorogenic acid and 200 mg/kg baicalin, respectively. The supplement was provided by Beijing Centre Biology Co., Ltd. (Beijing, China).

Five-tier ladder cages, with a capacity of four birds per cage, were used to house all the hens. The photoperiod was set at 16 h of light per day, and the room temperature was maintained at 20 ± 3 °C throughout the 10-week study. The birds had unrestricted access to food and water, and eggs were collected daily at 4 pm.

### 2.2. Laying Performance Measurement

The total number of eggs and the egg weight per replicate were recorded on a daily basis. Weekly records were kept of residual feed consumption. At the end of week 75 and 80, laying rate, average egg weight, average daily feed intake (ADFI) and feed/egg ratio were calculated. All calculations are based on the above records.

### 2.3. Sample Collection and Preparation

At the conclusion of the experiment, two birds with comparable body weight were selected from each replicate. After an 8-h fasting period, the birds were weighed, and blood samples were drawn from the wing vein into vacuum tubes designed to promote coagulation. These blood samples were kept at room temperature for 4 h, after which serum was obtained by centrifuging the samples at 3000 rpm for 15 min at 4 °C. Subsequently, the hens were anesthetized using sodium pentobarbital and euthanized via exsanguination from the jugular vein. Follicles were classified according to their diameter as either pre-grade or preovulatory follicles (≥12 mm) [13]. Pre-grade follicles were further categorized into small white follicles (SWF, 1–2 mm), large white follicles (LWF, 2–6 mm), small yellow follicles (SYF, 6–8 mm), and large yellow follicles (LYF, 8–12 mm) [14]. These follicles were identified and counted. Ovaries lacking follicles larger than 1 mm in diameter were weighed to determine the ovarian index (g/kg), calculated as the ovary weight (g) divided by the bird’s body weight (kg). Aseptic collection of cecal contents was performed for each bird. Serum, ovaries (excluding follicles larger than 1 mm), and cecal samples were then stored at −80 °C for subsequent analysis.

### 2.4. Antioxidant Indices

The kits used for the determination of glutathione peroxidase (GSH-Px), total superoxide dismutase (T-SOD), catalase (CAT), total antioxidant capacity (T-AOC) and malondialdehyde (MDA) in the serum and ovary were purchased from Nanjing Jiancheng Bioengineering Institute (Nanjing, China). The procedures were carried out as previously described [15].

### 2.5. Immune Parameters

Immunoglobulins Y (IgY), immunoglobulins A (IgA) and immunoglobulins M (IgM) concentrations in the serum were determined using commercially available chicken specific enzyme-linked immunosorbent assay (ELISA) kits (Nanjing Jiancheng Bioengineering Institute, Nanjing, China). The experimental approach was based on the protocol described by Wattrang E et al. [16].

Interleukin-6 (IL-6), tumor necrosis factor-alpha (TNF-α) and interferon-γ (IFN-γ) kits were purchased from Cloud-Clone Corp. (Wuhan, China), and interleukin-1β (IL-1β) kit was purchased from Cusabio Biotech Co., Ltd. (Wuhan, China). The methodology followed that described in earlier publication [17].

### 2.6. Hormonal Assays

Estradiol (E_2_), follicle-stimulating hormone (FSH) and luteinizing hormone (LH) levels in the serum were measured using commercial ELISA kits purchased from Nanjing Jiancheng Bioengineering Institute (Nanjing China). The procedures were carried out in the same way as they were previously described [18].

### 2.7. 16S rRNA Gene Sequencing of Cecal Microbiota

Microbia DNA was extracted from the cecal content using the DNAiso Reagent (TAKARA, Kyoto, Japan). The quality of extracted DNA was checked with gel electrophoresis. TheV3-V4 hypervariable regions of bacterial 16S rRNA were amplified using universal primer pairs (338F: 5′-ACTCCTACGGAGGCACAG-3′; 806R: 5′-GGACTACHVGGGTWTCTAAT-3′). Pair sequencing was performed on an Illumina HiSeq2500 PE300 platform (Illumina, San Diego, CA, USA) at Majorbio Bio-Pharm Technology Co., Ltd. (Shanghai, China). The raw paired-end reads were submitted to the Trimmomatic software (version 0.39) for quality filtering and then merged using FLASH 1.2.7 (https://ccb.jhu.edu/software/FLASH/index.shtml, accessed on 6 September 2024). Clustering of Amplicon Sequence Varian (ASV) with the requirement of 100% similarity by means of UPARSE (version 7.1, http://drive5.com/uparse/, accessed on 6 September 2024). The taxonomy of each 16S rRNA gene sequence was analyzed using the RDP classifier algorithm with a confidence threshold of 70%. Data analysis was performed using the Majorbio Cloud Platform (Majorbio Bio-Pharm Technology Co., Ltd., Shanghai, China). The Ace, Chao and Shannon indices were calculated using Mothur-1.30.2. To estimate pairwise distances between samples and to determine β-diversity, the Principal Coordinates Analysis (PCoA) plot based on unweighted Unifrac was used. Linear discriminant analysis (LDA) coupled with effect size measurements (LEfSe) were used to identify the microbial taxa biomarkers between groups, with a selection criterion of an LDA score greater than 2.0. Wilcoxon rank-sum test was used to evaluate the functional differences between the CON and VE groups.

### 2.8. Untargeted Metabolomics Analysis of Ovary

A total of 12 ovarian samples, each weighing 20 ± 1 mg, were obtained for metabolomic analysis, with 6 samples from the CON group and 6 from the PE group. Metabolites were extracted from the samples using methanol/water (70%, *v*/*v*) and 2-chlorophenylalanine (1 μg/mL). A 200 μL supernatant was collected in a liquid chromatographic bottle for LC-MS/MS analysis after centrifugation (4 °C, 12,000 rpm, 10 min). The extraction solution of all samples in the same group is mixed in equal volumes for the preparation of the quality control sample. Metabolite samples were analyzed using a UHPLC system (LC20, Shimadzu, Kyoto, Japan) coupled with an ACQUITY UPLC HSS T3 C18 column (1.8 µm, 2.1 mm × 100 mm; Waters Corp., Milford, MA, USA). The mobile phases consisted of ultrapure water with 0.1% formic acid (A) and acetonitrile with 0.1% formic acid (B). The elution gradient was as follows: 95% A and 5% B (*v*/*v*) at 0 min; 10% A and 90% B (*v*/*v*) at 11.0 min; 10% A and 90% B (*v*/*v*) at 12.0 min; 95% A and 5% B (*v*/*v*) at 12.1 min; 95% A and 5% B (*v*/*v*) at 14.0 min. The column temperature was maintained at 40 °C, with a 2 µL injection volume and a flow rate of 0.4 mL/min. Mass spectrometric detection was performed using both positive and negative ion modes on the Triple TOF-6600 mass spectrometer (AB SCIEX, Framingham, MA, USA).

Data analysis was performed using the self-built MWDB database (Metware Biotechnology Co., Ltd., Hubei, Wuhan, China). Metabolites exhibiting a variable importance in projection (VIP) greater than 1, along with a fold change (FC) exceeding 2 or less than 0.05, were utilized as criteria for the identification of potential biomarkers. The KEGG pathway analysis was used to conduct metabolite pathway enrichment analysis on the differential metabolites to determine the differential metabolic pathways between the CON and PE groups.

### 2.9. Statistical Analysis

Student’s *t*-test was used to analyze the data (expected for gut microbes and ovarian metabolites). Results were presented as “mean ± standard deviation.” A significance level of *p* < 0.05 was established for statistical analysis. Spearman correlation analysis was carried out to explore the intricate relationships among gut microbes (at the genus level), the significantly identified ovarian metabolites, immunoglobulins, antioxidant parameters, and performance-related parameters. GraphPad Prism 8.0 (GraphPad Software Inc., San Diego, CA, USA) was used to create the figures.

## 3. Results

### 3.1. Laying Performance

As shown in Table 1, the PE group exhibited a notable (*p* < 0.05) improvement in laying rates (6.36%, 9.27%, 8.12%) compared to the CON group, while also showing a reduction in feed/egg ratio (7.42%, 9.43%, 8.8%) across weeks 71 to 75, 76 to 80, and 71 to 80. However, no significant differences (*p* > 0.05) were observed between the CON and PE groups regarding average egg weight and average daily feed intake (ADFI) during the same periods.

### 3.2. Antioxidant Parameters

**Figure 1:**
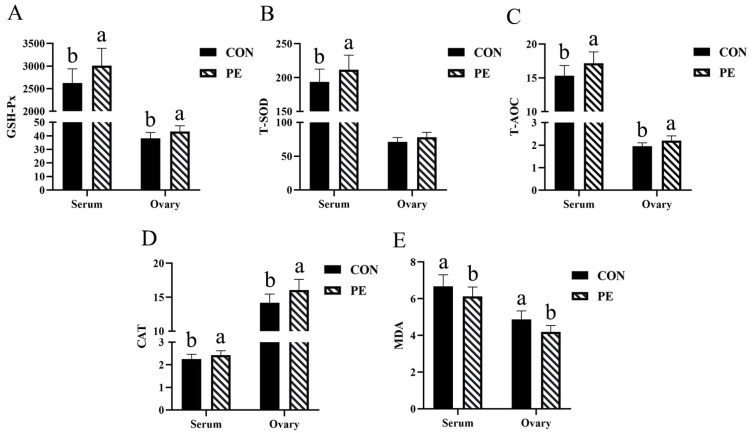
Effect of PE on antioxidant parameters of laying hens. Note: CON, control group; PE, plant extracts of *Flos lonicerae* and *Baikal skullcap* group. The data are expressed as the mean ± standard deviation (*n* = 12 per group). Statistical significance (*p* < 0.05) is indicated by different letters in the bar chart. (**A**) GSH-Px: glutathione peroxidase. (**B**) T-SOD: total superoxidedismutase. (**C**) T-AOC, total antioxidant capacity. (**D**) CAT, catalase; (**E**) MDA: malondialdehyde. The GSH-Px, T-SOD, T-AOC and CAT were expressed as specific activity (U/mgprot) in ovary and as (U/mL) in serum, respectively. The MDA was expressed as (nmol/mgprot) in ovary and as (nmol/mL) in serum, respectively.

The data presented in Figure 1 demonstrate that the antioxidant capacity of hens in the PE group was generally superior to that observed in the CON group. This was supported by the following findings: a significant increase (*p* < 0.05) in serum T-SOD activity (9.37%); elevated (*p* < 0.05) levels of GSH-Px (14.62% and 13.40%), T-AOC (12.14% and 12.78%), and CAT (7.52% and 13.26%) in both serum and ovarian tissues; and a notable reduction (*p* < 0.05) in the concentrations of MDA in both serum (8.10%) and ovary (13.96%).

### 3.3. Cytokine Levels

**Figure 2:**
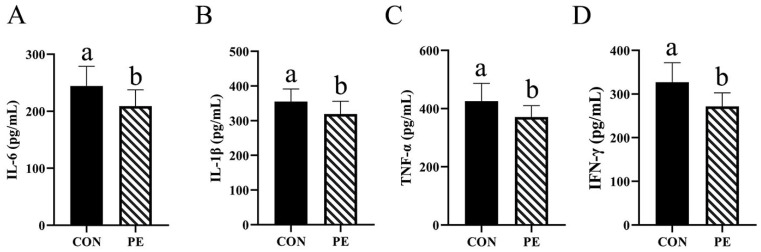
Effect of PE on the level of cytokine. Note: CON, control group; PE, plant extracts of Flos lonicerae and Baikal skullcap group. The data are expressed as the mean ± standard deviation (*n* = 12 per group). Statistical significance (*p* < 0.05) is indicated by different letters in the bar chart. (**A**) IL-6: Interleukin-6. (**B**) IL-1β: interleukin-1β. (**C**) TNF-α, tumor necrosis factor-alpha. (**D**) IFN-γ: interferon-γ. The IL-6, IL-1β, TNF-α and IFN-γ were expressed as (pg/mL) in serum.

As illustrated in Figure 2, the PE group exhibited a statistically significant reduction (*p* < 0.05) in serum levels of IL-6, IL-1β, TNF-α and IFN-γ, with reductions of 14.34%, 10.07%, 12.72% and 16.84%, respectively, in comparison to the CON group (Figure 2A–D).

### 3.4. Immune Indices

**Figure 3:**
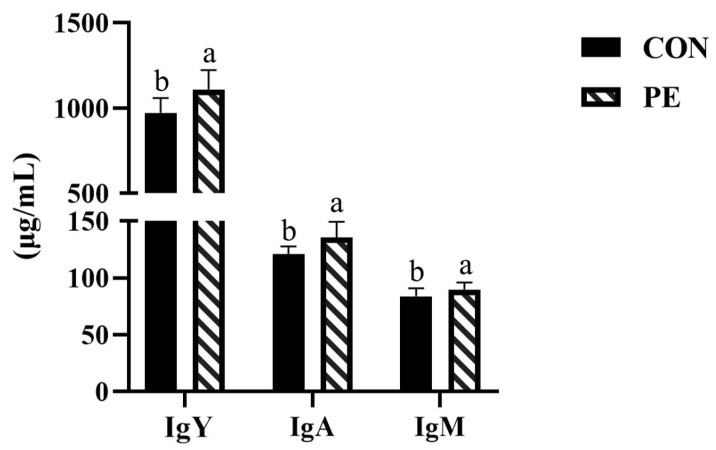
Effect of PE on serum immune indexes of laying hens. Note: CON, control group; PE, plant extracts of *Flos lonicerae* and *Baikal skullcap* group. Data were presented as mean ± standard deviation (*n* = 12 per group). Different superscript letters in the bar chart indicate significant differences (*p* < 0.05). IgA, immunoglobulin A; IgM, immunoglobulin M; IgY, immunoglobulin Y.

According to Figure 3, we found that PE group increased (*p* < 0.05) IgY, IgA and IgM levels in serum by 14.06%, 11.74% and 6.82%, respectively, compared with CON group.

### 3.5. Ovary Index and Follicles Numbers

**Figure 4:**
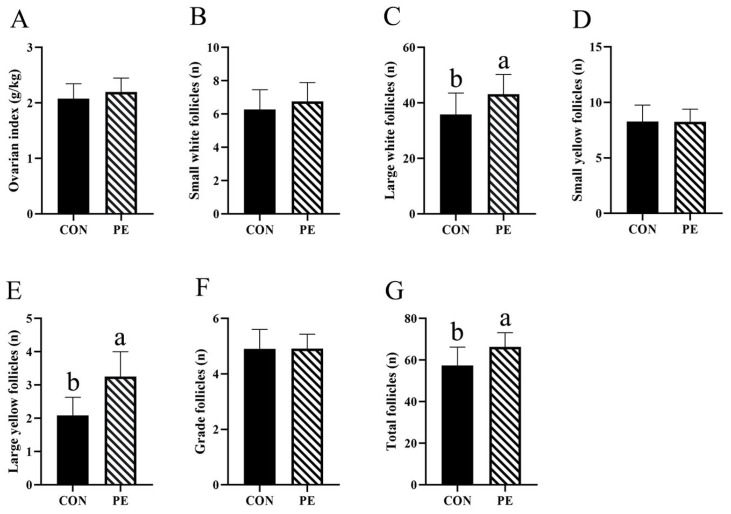
Effect of PE on the number of follicles. Note: CON, control group; PE, plant extracts of *Flos lonicerae* and *Baikal skullcap* group. The data are expressed as the mean ± standard deviation (*n* = 12 per group). Statistical significance (*p* < 0.05) is indicated by different letters in the bar chart. (**A**) Ovarian index; (**B**) The number of small white follicles; (**C**) The number of large white follicles; (**D**) The number of small yellow follicles; (**E**) The number of large yellow follicles; (**F**) The number of grade follicles; (**G**) The number of grade follicles total follicles.

The results for ovary index and follicle numbers are shown in Figure 4. In comparison to the CON group, the PE group exhibited significant increases (*p* < 0.05) SWF (7.66%), LWY (20.52%), LYF (55.50%) and total follicles numbers (15.56%), as well as a rise in ovary index (6.28%) (Figure 4A–C,E,G). Nevertheless, the numbers of SYF and grade follicles were not significantly altered by PE supplementation (Figure 4D,F).

### 3.6. Serum Estrogen Levels

**Figure 5:**
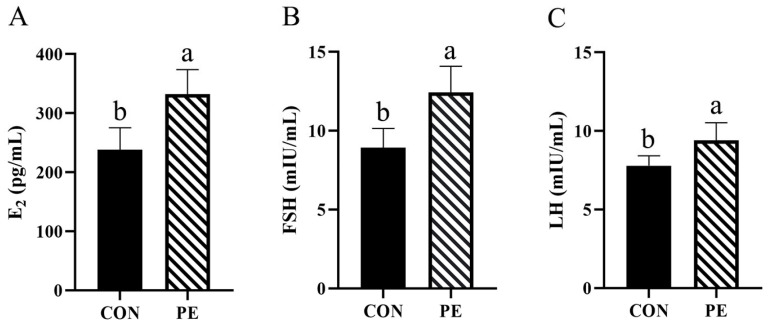
Effect of PE on serum estrogen levels of laying hens. Note: CON, control group; PE, plant extracts of *Flos lonicerae* and *Baikal skullcap* group. The data are expressed as the mean ± standard deviation (*n* = 12 per group). Statistical significance (*p* < 0.05) is indicated by different letters in the bar chart. (**A**) E_2_: estradiol; (**B**) FSH: follicle-stimulating hormone; (**C**) LH: luteinizing hormone.

The results presented in Figure 5 show that PE supplementation significantly (*p* < 0.05) increased serum FSH (38.99%), E_2_ (39.16%) and LH (20.92%) levels (Figure 5A–C).

### 3.7. Cecal Microbiota

**Figure 6:**
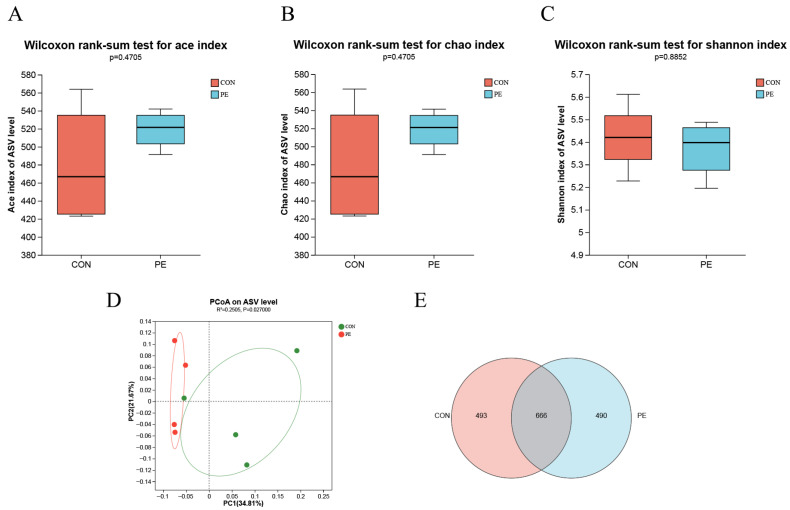
An analysis of microbial diversity in the cecal contents was conducted between the PE and CON groups. The α-diversity comparison between the PE and CON groups was assessed using the Ace index (**A**), Chao index (**B**) and Shannon index (**C**). (**D**) β-diversity between PE and CON groups display as principal coordinates analysis. (**E**) Venn diagram of two microbial communities based on ASV. CON, control group; PE, plant extracts of *Flos lonicerae* and *Baikal skullcap* group. Data are expressed as mean ± standard deviation (*n* = 4 per group).

The Ace and Chao indices are used to measure the community richness of samples, while the Shannon index serves to assess the community diversity. The results indicated that the Ace and Chao indices, which represent α-diversity, were significantly greater (*p* < 0.05) in the PE group compared to the CON group (Figure 6A,B). There were no apparent alterations (*p* > 0.05) in the Shannon index (also representative of α-diversity) between the PE and CON groups (Figure 6C). To assess the similarity in microbial community structure (β-diversity) between the PE and CON groups, we performed principal coordinate analysis (PCoA) (Figure 6D). The PCoA results demonstrated a distinct separation of microbial communities between the PE and CON groups. The microbial communities were clearly clustered, allowing for the clear division of the data into two distinct groups (Figure 6D). As demonstrated in Figure 6E, the cecal microbiotas of hens in the PE and CON groups exhibited significant overlap, with the two groups sharing a total of 666 OTUs. The birds from the CON and PE groups exhibited 493 and 490 specific OTUs, respectively (Figure 6E).

We examined the alterations in microbiota composition at the genus level (Table 2). The *Bacteroides*, *Phascolarctobacterium* and *unclassified_f__Lachnospiraceae* accounted for the largest proportion of the microbiota. Compared with CON group, PE group increased (*p* < 0.05) *Bacteroides* and *Butyricicoccus* abundance. Marked upregulation of *Phascolarctobacterium*, *unclassified_f__Lachnospiraceae*, *Ruminococcus_torques_group*, *norank_f__norank_o__Clostridia_UCG-014*, *norank_f__Eubacterium_coprostanoligenes_group*, *Lactobacillus*, *Christensenellaceae_R-7_group* and *Colidextribacter*, along with a downregulation in the abundance of *Prevotellaceae_UCG-001*, *Faecalibacterium*, *Desulfovibrio*, *Subdoligranulum*, *Megamonas*, *norank_f__Muribaculaceae*, *Romboutsia*, *Alistipes* and *norank_f__Prevotellaceae*. However, these changes were not statistically significant (*p* > 0.05).

**Figure 7:**
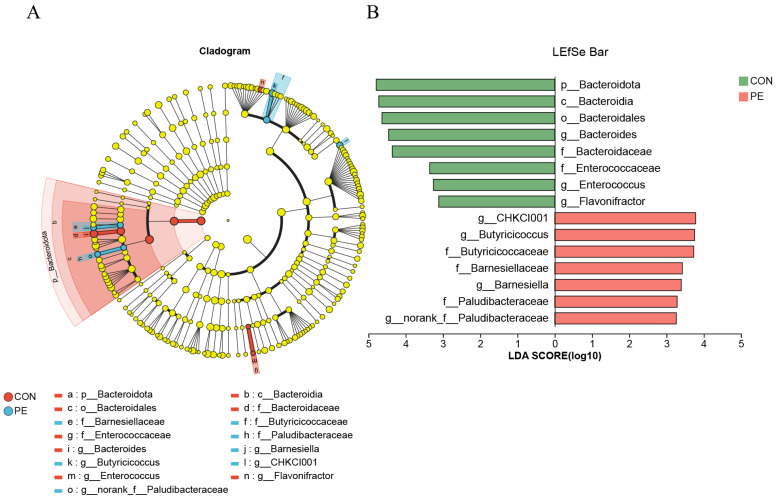
Effect size measurements analysis of cecal microbiota. Note: CON, control group; PE, plant extracts of *Flos lonicerae* and *Baikal skullcap* group. (**A**) Effect size measurements multi-level species hierarchy tree diagram. (**B**) Taxonomic biomarkers with linear discriminant analysis (LDA) score (log10) > 4. The LDA score is represented by the length of the histogram.

The LEfSe analysis revealed that the taxonomic markers identified in the PE group included *Barnesiellaceae* (family level), *Butyricicoccaceae* (family level), *Paludibacteraceae* (family level), *Barnesiella* (genus level), *Butyricicoccus* (genus level), *CHKCI001* (genus level), and *norank_f__Paludibacteraceae* (genus level). In contrast, the taxonomic markers found in the CON group encompassed *Bacteroidota* (phylum level), *Bacteroidia* (class level), *Bacteroidales* (order level), *Bacteroidaceae* (family level), *Enterococcaceae* (family level), *Bacteroides* (genus level), and *Enterococcus* (genus level) (Figure 7).

### 3.8. Ovary Metabolome

**Figure 8:**
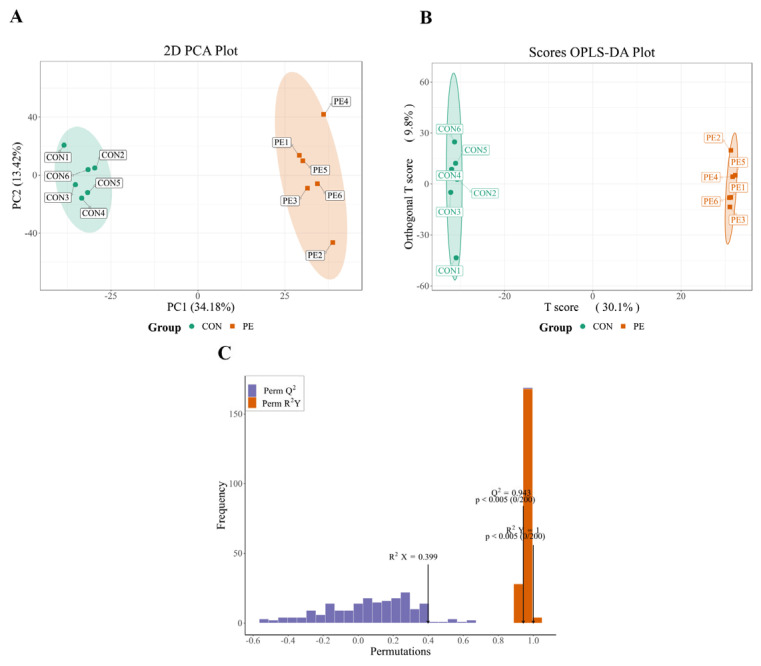
Untargeted ovary metabolomics multivariate analysis. Note: CON, control group; PE, plant extracts of *Flos lonicerae* and *Baikal skullcap* group. (**A**) The principal component analysis (PCA) scatter plot (*n* = 6 per group) illustrates the variation in metabolites between the CON and PE groups. (**B**) The partial least squares discriminant analysis (PLS-DA) score plot (*n* = 6 per group) highlights the metabolic distinctions separating the CON and PE groups. (**C**) The PLS-DA validation plot presents the R^2^ and Q^2^ parameters, which serve to confirm the robustness of the model.

The metabolic patterns of the CON group were clearly separated from those of the PE group based on OPLS-DA and PCA analyses (Figure 8A,B), suggesting that PE supplementation induced notable alterations in ovarian metabolites. The OPLS-DA score plot further demonstrated strong model performance, with values of R^2^Y = 1 and Q^2^ = 0.943, confirming the robustness and reliability of the model (Figure 8C).

Furthermore, as presented in Table 3, a total of 17 metabolites (both upregulated and downregulated) were identified as potential biomarkers, based on the screening criteria of *p* < 0.01 and FC ≥ 20 or FC ≤ 0.01. Relative to the CON group, the levels of Leu-Enkephalin (L-ENK), palmitoyl serotonin, repaglinide, 2′-Hydroxy-4,4′,6′-trimethoxychalcone, fenaminosulf, lasalocid, 2-methoxyestradiol (2ME), amsacrine, vanillin, nicotinic acid adenine dinucleotide (NAD), carboplatin, enalapril, bosentan, rosuvastatin were markedly elevated in the PE group (*p* < 0.01). In contrast, the concentrations of urobilin, paxilline, and hexaconazole were significantly reduced in the PE group (*p* < 0.01).

**Figure 9:**
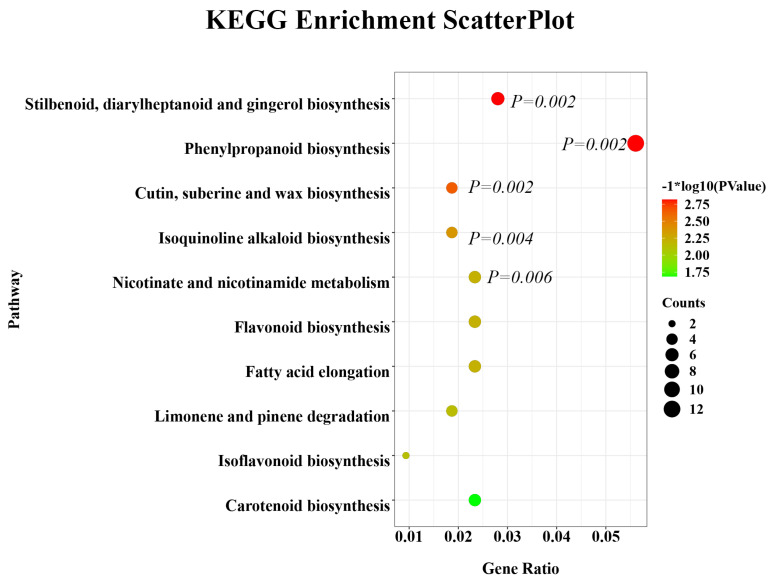
Analysis of KEGG pathways.

To explore the potential metabolic pathways, KEGG enrichment analysis was conducted on the metabolites that differed significantly between the CON and PE groups. As illustrated in Figure 9, supplementation with PE was primarily associated with modifications in stilbenoid, diarylheptanoid, and gingerol biosynthesis (*p* = 0.002), followed by phenylpropanoid biosynthesis (*p* = 0.002), cutin, suberine and wax biosynthesis (*p* = 0.002), isoquinoline alkaloid biosynthesis (*p* = 0.004) and nicotinate and nicotinamide (NAM) metabolism (*p* = 0.006).

### 3.9. Spearman Correlation Analysis

Spearman’s correlation analysis was conducted to investigate the potential associations among the significantly altered metabolites, specific gut microbial populations, production traits, cytokine concentrations, as well as antioxidant and immune indices.

**Figure 10:**
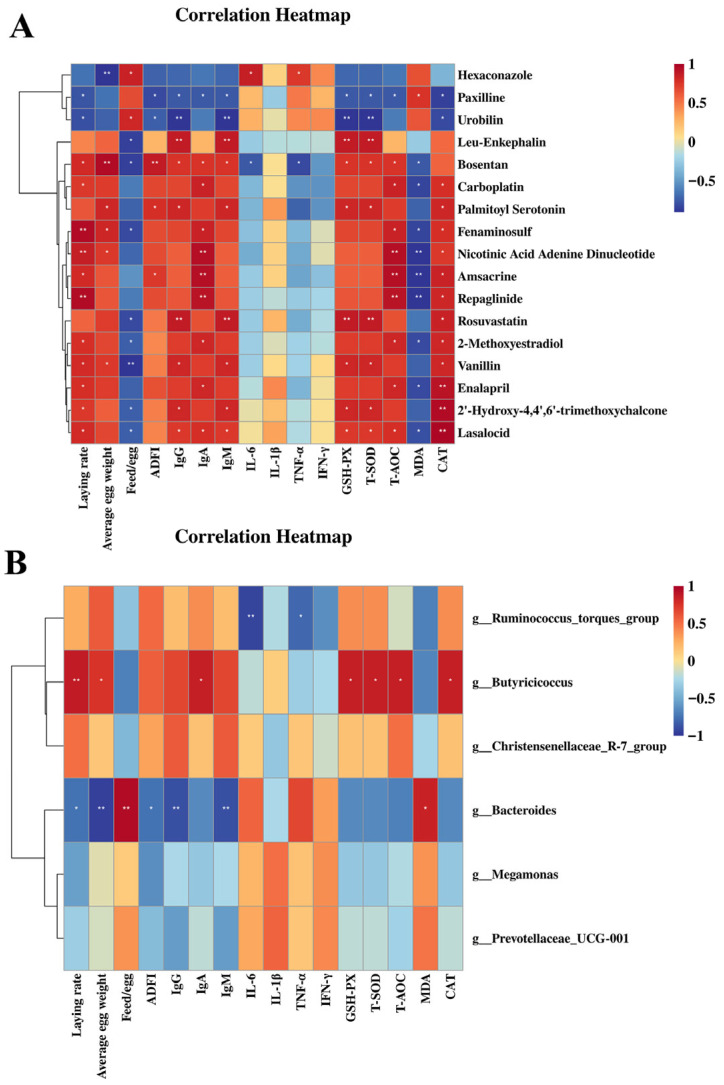
Correlation analysis of ovary metabolites and gut microbes (at genus level) with immune, antioxidant and performance parameters. Note: positive and negative correlations are shown in red and blue panels (color intensity indicates Spearman’s r-value of the correlation in each panel). * *p*< 0.05, ** *p*< 0.01. (**A**) The correlation of significantly changed ovary metabolites with immunoglobulins, ovary antioxidant parameters and performance parameters. (**B**) The correlation of gut microbes (at genus level) with immunoglobulins, serum antioxidant parameters and performance parameters.

As represented in Figure 10A. Urobilin and paxilline were negatively correlated with laying rate, immunoglobulins (particularly IgY and IgM) and ovary antioxidant parameters (particularly GSH-Px and T-SOD) (*p* < 0.05). Hexaconazole was negatively correlated with average egg weight (*p* < 0.01) and IL-6 and IFN-γ (*p* < 0.05). Fenaminosulf, NAD and repaglinide were positively correlated with laying rate (*p* < 0.01). Bosentan was positively correlated with average egg weight and ADFI (*p* < 0.01). L-ENK, NAD, amsacrine, repaglinide and rosuvastatin were positively correlated with immunoglobulins and ovary antioxidant parameters (particularly GSH-Px, T-SOD, T-AOC and MDA) (*p* < 0.05). Enalapril, 2′-Hydroxy-4,4′,6′-trimethoxychalcone and lasalocid were positively correlated with ovary antioxidant parameters (particularly CAT) (*p* < 0.05).

As shown in Figure 10B, *Butyricicoccus* (at genus level) showed a significant positive correlation with laying rate (*p* < 0.01), average egg weight (*p* < 0.05), IgA (*p* < 0.05) and serum GSH-Px, T-SOD, T-AOC and CAT (*p* < 0.05). *Bacteroides* (at genus level) was negatively correlated with laying rate, ADFI (*p* < 0.05), average egg weight (*p* < 0.01), IgY and IgM (*p* < 0.05), and positively correlated with the feed/egg ratio (*p* < 0.01) and serum MDA (*p* < 0.05). *Ruminococcus_torques_group* showed a significant positive correlation with IL-1β (*p* < 0.01) and IFN-γ (*p* < 0.05).

## 4. Discussion

The results of this experiment indicate that the addition of 500 mg/kg PE to the diet increased the laying rate and feed conversion efficiency of laying hens from 71 to 75 weeks, 76 to 80 weeks and 71 to 80 weeks, which were basically consistent with previous report [8]. Wang et al. [8] reported that dietary supplementation with 1000 mg/kg of PE enhanced egg production in 41-week-old Jinghong laying hens. While the improvement in laying rate has often been attributed to the antioxidant, antibacterial, anti-inflammatory, and antiviral properties of CGA and baicalin [19,20], our metabolomic results provide more specific insights. PE supplementation significantly altered stilbenoid, diarylheptanoid, gingerol, and phenylpropanoid biosynthesis, which generate bioactive metabolites with estrogen-like and anti-inflammatory activities. These metabolites could enhance ovarian sensitivity to estrogen and reduce follicular atresia, thereby supporting sustained egg production in aged hens. Additionally, the enrichment of nicotinate and nicotinamide metabolism may regulate mitochondrial redox balance and energy metabolism, further contributing to improved feed efficiency.

The main active components of *Flos lonicerae* and *Baikal skullcap* extract are CGA and baicalin, both possessing phenolic hydroxyl groups that scavenge free radicals and exert strong antioxidant effects [21,22]. Study of Chen et al. [23] revealed that diets supplemented with 0.2%, 0.4% and 0.8% *Flos lonicerae* increased the GSH-Px activities and decreased the MDA contents in the hepatopancreas of Penaeus monodon. Analogously, Liao et al. [24] confirmed that dietary supplementation with 60, 120, 180 or 240 mg/kg *Baikal skullcap* increased liver T-SOD and GSH-Px activities in 42 d Arbor Acres male broilers. Consistently, our study found that 500 mg/kg PE supplementation increased serum and ovarian CAT, T-AOC, T-SOD, and GSH-Px levels, while reducing MDA concentrations in the serum and ovary, indicating that PE addition may improve ovarian antioxidant status of aged laying hens.

Immunoglobulins (IgM, IgA, IgY) are key indicators of humoral immunity [25]. In this study, dietary supplementation with 500 mg/kg PE significantly increased serum IgM, IgA, and IgY levels in late-phase laying hens, suggesting enhanced immune function. These results are consistent with Wang et al. [8], who observed a significant increase in serum IgM levels in 41-week-old Jinghong laying hens that were challenged with *Salmonella pullorum*, following dietary supplementation of 1000 mg/kg PE. Although the improved immunity has been previously attributed to protection of immune cells from oxidative stress [23], our data suggest additional mechanisms. For instance, nicotinate and nicotinamide metabolism may enhance NAD^+^-dependent signaling pathways that regulate immune cell activation. Moreover, PE supplementation increased the abundance of *Butyricicoccus*, which was positively correlated with IgA levels and antioxidant enzyme activities. This genus is known to produce short-chain fatty acids (SCFAs) that modulate gut-immune interactions, thereby providing a plausible explanation for the enhanced immune response observed in PE-treated hens.

During the late laying period, excessive fat deposition can induce oxidative stress and stimulate pro-inflammatory cytokine release [26,27,28]. PE supplementation enhanced the antioxidant capacity of laying hens, suggesting more effective ROS scavenging and a potential reduction in pro-inflammatory factors. This effect is consistent with the reported anti-inflammatory properties of CGA and baicalin, which can inhibit the secretion of pro-inflammatory factors, particularly TNF-α, IL-1β and IL-6 [29,30], further supporting the potential immunomodulatory role of PE. Importantly, specific metabolites elevated by PE, such as 2-methoxyestradiol and vanillin, are known to suppress NF-κB–mediated transcription of pro-inflammatory cytokines. Additionally, the enrichment of gingerol biosynthesis, which produces compounds that inhibit IL-1, TNF-α, and IL-8 synthesis [31]. As expected, the levels of TNF-α, IFN-γ, IL-1β and IL-6inthe serum were higher in the CON group than those in the PE group in this study, which were in line with previous study of Ishfaq et al. [32].

The ovary plays a vital role in reproduction, with ovarian index and follicle number positively correlated with laying rate [33,34]. In our study, 500 mg/kg PE supplementation increased ovarian index and follicle numbers (SWF, LWF, LYF). High egg production is linked to reduced follicular atresia [35], whereas oxidative stress accelerates granulosa cell apoptosis and corpus luteum degeneration, leading to follicular loss [36]. In this study, PE supplementation improved antioxidant and immune functions in laying hens, thereby alleviating oxidative stress-induced follicular atresia and increasing the number of different follicular patterns. Estrogen promotes the development of ovarian follicles by helping the granulosa cells to proliferate [37]. During the late laying period of hens, estrogen secretion decreases as ovarian function declines, resulting in an increase in follicular atresia and a decrease in the number of developing follicles [38]. Our findings revealed that PE supplementation significantly enhanced serum E_2_, FSH and LH levels. This improvement may be attributed to the potential role of PE in enhancing ovarian antioxidant capacity and attenuating the progression of ovarian aging. In this study, the capacity of PE to elevate estrogen levels could potentially account for the enhanced number of different follicular patterns (SWF, LWF and LYF) in the PE group.

The gut microbiota strongly influences nutrient utilization and host immunity [39]. In the current study, dietary supplementation with 500 mg/kg PE increased the α-diversity of the cecal microbial community, as reflected by higher Ace and Chao indices. The possible reason for this phenomenon is that PE may enable certain indigenous bacteria to become dominant bacteria, which in turn optimizes the original microbial community structure, leading to an increase in microbial community diversity. However, the specific mechanism still needs to be investigated in more detail. Notably, the relative abundance of *Ruminococcus_torques_group*, *Butyricicoccus*, and *Christensenellaceae_R-7_group* at the genus level was elevated in the PE group. Both *Ruminococcus_torques_group* and *Butyricicoccus* are reported butyrate producers [40,41]. Butyrate contributes to intestinal health by promoting villus growth [42], supporting the balance of gut microbiota through stimulation of beneficial bacteria and inhibition of harmful bacteria [43], providing the majority of energy required for colonic epithelial metabolism, and enhancing intestinal mucin protein secretion [44]. Therefore, an increase in *Ruminococcus_torques_group* and *Butyricicoccus* abundances in the PE group meant that PE may play a crucial role in maintaining the normal physiological functions of the intestine. However, it should be noted that the present study did not directly measure butyrate levels in the small intestine, and most butyrate produced in the cecum is utilized locally by colonocytes. Therefore, additional analyses of butyrate levels in the small intestine and intestinal transport would be required to confirm whether cecal butyrate reaches the small intestine and exert systemic or extra-cecal effects. *Christensenellaceae_R-7_group*, which possess cellulase and hemicellulase genes, enhance ruminants’ ability to degrade polysaccharides and extract energy [45], and are positively correlated with the average daily gain of Yak [46]. Hence, our results suggest that PE may improve feed conversion efficiency and production performance of laying hens, which is consistent with the results of laying rate and feed/egg ratio in this experiment. In this study, correlation analysis showing that *Butyricicoccus* was positively associated with serum antioxidant and immune parameters as well as laying rate, while *Ruminococcus_torques_group* and *Christensenellaceae_R-7_group* displayed positive, though not statistically significant, correlations with these traits. In addition, *Ruminococcus_torques_group* and *Butyricicoccus* showed a negative correlation with serum pro-inflammatory factors. Therefore, we propose that the microbiota modified by PE supplementation played a crucial role in enhancing antioxidant and immune functions, maintaining intestinal health, and alleviating inflammatory responses, which ultimately leads to an improvement in the laying rate and feed conversion efficiency of laying hens. LEfSe analysis showed *Butyricicoccus* (at genus level) dominated in the PE group, consistent with overall microbial abundance results.

Furthermore, PE supplementation reduced the abundance of *Bacteroides*, *Prevotellaceae_UCG-001* and *Megamonas* at the genus level. *Bacteroides*, *Prevotellaceae_UCG-001* and *Megamonas* are generally involved in SCFAs production [47,48,49]. SCFAs, serving as an energy source for colonocytes, play a crucial role in modulating immune responses, maintaining intestinal barrier integrity, and regulating systemic metabolic pathways [50]. However, our study showed that *Bacteroides*, *Prevotellaceae_UCG-001* and *Megamonas* were negatively correlated with serum antioxidant and immune parameters as well as laying rate. *Bacteroides*, *Prevotellaceae_UCG-001* and *Megamonas* are generally important for host health; however, certain species within these genera can contribute to disease. *Bacteroides fragilis* is a recognized opportunistic pathogen, promoting chronic inflammation and producing enterotoxins that induce secretory diarrhea and colitis [51,52]. Similarly, *Megamonas hypermegale* and *Megamonas funiformis* have been linked to elevated inflammatory biomarkers in individuals with metabolic syndrome [53]. *Prevotella* is associated with intestinal inflammation and mucosal dysfunction and tends to accumulate in the mucosal tissue of ulcerative colitis patients [54,55]. In our study, PE supplementation reduced the abundance of *Bacteroides, Megamonas* and *Prevotellaceae_UCG-001*, suggesting a potential mitigation of inflammatory responses in laying hens. This interpretation was supported by Spearman correlation analysis, which showed positive associations between these genera and serum pro-inflammatory factors.

Metabolomics is a powerful tool for profiling metabolites in biological samples [56]. In this study, ovarian metabolomic analysis showed that multiple metabolites, including L-ENK, Palmitoyl Serotonin, Repaglinide, 2′-Hydroxy-4,4′,6′-trimethoxychalcone, Fenaminosulf, Lasalocid, 2ME, Amsacrine, Vanillin, NAD, Carboplatin, Enalapril, Bosentan and Rosuvastatin were elevated in the PE group compared with the CON group.

Hexaconazole has been reported to disrupt estrogen synthesis by interfering with steroidogenic enzymes and aromatase activity [57,58,59], whereas L-ENK acts as a regulator of female reproduction via the hypothalamic-pituitary-ovarian axis (HPOA) [60]. Ovarian index and oocyte diameter were significantly increased in shrimp (*Penaeus indicus*) after injection of L-ENK [61]. In addition, by regulating HPOA, L-ENK was observed to stimulate oogonia proliferation in *Oreochromis mossambicus* [62]. In this study, L-ENK was positively correlated with laying rate, while hexaconazole showed the opposite trend, suggesting that PE may enhance egg production by increasing ovarian L-ENK and reducing ovarian hexaconazole levels. This aligns with previous evidence that baicalin promotes steroid hormone production in granulosa cells of CD-1 mice [63].

2ME exhibits both anti-inflammatory and antioxidant activities, suppressing pro-inflammatory cytokines production and ROS-induced proliferation and migration of vascular smooth muscle cells [64,65]. Similarly, vanillin can neutralize ROS via self-polymerization and reduce inflammatory cytokine expression in mice [66,67]. Enalapril has been shown to eliminate oxidative stress in rodent models [68,69]. Age-related factors such as oxidative stress and mitochondrial activity are profoundly affected by NAD [70]. Many models have suggested that a decline in NAD levels is a hallmark of aging [71]. Our results showed that 2ME, vanillin, enalapril and NAD were positively associated with immune and ovarian antioxidant parameters as well as laying rate, suggesting that PE may improve the laying rate by improving immune and anti-inflammatory functions as well as ovarian antioxidant capacity.

Fenaminosulf, a common fungicide [72], and lasalocid, used against coccidiosis [73], were positively correlated with immune and ovarian antioxidant parameters. Therefore, the increase in fenaminosulf and lasalocid in the PE group suggests PE may exert antibiotic-like effects.

Vanillin is an important flavor ingredient used worldwide [67]. Our results suggest that PE may have a role in improving the aroma of egg yolks. Rosuvastatin lowers cholesterol synthesis in the liver [74]. Our findings suggest that PE may reduce cholesterol levels in egg yolks and plasma, consistent with CGA’s cholesterol-lowering effects in rats [75]. Moreover, as reduced urobilin levels are linked to better liver health [76], our findings suggest that PE might exert hepatoprotective effects, which may ultimately enhance the laying rate.

KEGG analysis showed that PE supplementation enhanced metabolic pathways, primarily stilbenoid, diarylheptanoid and gingerol biosynthesis as well as phenylpropanoid biosynthesis, and secondarily cutin, suberine and wax biosynthesis, isoquinoline alkaloid biosynthesis as well as nicotinate and NAM metabolism.

Stilbenoid, diarylheptanoid and gingerol possess significant medicinal value due to their anticancer, antioxidant and anti-inflammatory properties [31,77,78]. Representative stilbenoid compounds such as resveratrol, pinosylvin, and pinosylvin monomethyl ether exhibit strong antioxidant and anti-inflammatory effects [79], and resveratrol has been shown to alleviate aflatoxin B_2_-induced oxidative stress in mice [80]. Diarylheptanoids display estrogen-like activity and can activate estrogen receptors in vivo [78], while gingerol, a major active component of ginger, possesses antioxidant capacity and inhibits the synthesis of pro-inflammatory cytokines such as IL-1, TNF-α and IL-8 [81]. In addition, phenylpropanoids are widely applied as antioxidants and anti-inflammatory or antimicrobial agents [82]. In our study, the enrichment of stilbenoid, diarylheptanoid and gingerol biosynthesis as well as phenylpropanoid biosynthesis in the PE group indicated that PE can not only enhance ovarian anti-inflammatory and antioxidant capacity, but also improve estrogen sensitivity, thereby delaying ovarian aging.

Isoquinoline alkaloids are an important class of alkaloids with diverse pharmacological activities such as antibacterial, antiviral, anti-parasitic, anti-inflammatory, antioxidant and anti-ulcer [83]. Ni et al. [84] reported that isoquinoline alkaloid can improve gut health and digestive function in swine and poultry. In our experiment, the isoquinoline alkaloid biosynthesis was enriched by PE supplementation, suggesting that PE may improve intestinal antioxidant, anti-inflammatory, digestive and absorptive functions, thus increasing laying rate and reducing feed/egg ratio in old laying hens. This aligns with Vieira et al. [85], who found that isoquinoline alkaloids supplementation improved growth performance and feed conversion rate in broiler chicks.

NAM is a key regulator of mitochondrial metabolism and redox balance, acting to inhibit protein oxidation and lipid peroxidation [86,87]. Kwak et al. [87] reported that NAM can significantly reduce ROS levels in both senescent cells and cells undergoing senescence, while external NAM supplementation may mitigate ROS-induced cellular damage [88]. Niacin contributes to mitochondrial health by forming NAD and NADP, maintaining mitochondrial integrity and promoting mitochondrial biogenesis. Study by Adebowale et al. [89] demonstrated that niacin supplementation improved the heterophil/lymphocyte ratio and leukocytes in Aviagen turkeys. The enrichment of nicotinate and NAM metabolism due to the PE increment revealed that PE may enhance ovarian antioxidant capacity and have a positive modulation of the immune system.

## 5. Conclusions

This study provides novel evidence that PE supplementation improves laying performance and ovarian function of aged hens by modulating antioxidant status, inflammatory responses, ovarian metabolites, and cecal microbiota. These findings highlight PE as a promising natural green feed additive with potential to extend the laying cycle of laying hens and enhance sustainability in the poultry industry. Future studies should explore how the identified key metabolites, which target the enhancement of ovarian function, influence the production performance of laying hens during the late laying period, thereby enabling precise modulation of ovarian aging.

## Figures and Tables

**Table 1:** Effect of PE increment on laying performance of laying hens.

Items	CON	PE	*p*-Value
71–75 W			
Initial laying rate (%)	71.26 ± 1.23	71.13 ± 0.93	0.835
Laying rate (%)	67.44 ± 2.26 ^b^	71.73 ± 2.85 ^a^	0.016
Average egg weight (g)	53.20 ± 0.84	54.13 ± 1.39	0.189
feed/egg ratio	2.83 ± 0.10 ^a^	2.62 ± 0.10 ^b^	0.005
ADFI (g)	102.29 ± 1.00	102.16 ± 1.48	0.863
76–80 W			
Laying rate (%)	68.72 ± 2.24 ^b^	75.09 ± 1.96 ^a^	<0.001
Average egg weight (g)	54.00 ± 0.95	55.12 ± 1.62	0.176
feed/egg ratio	2.65 ± 0.06 ^a^	2.40 ± 0.10 ^b^	<0.001
ADFI (g)	98.14 ± 1.66	99.33 ± 1.24	0.190
71–80 W			
Laying rate (%)	68.20 ± 2.20 ^b^	73.74 ± 2.00 ^a^	0.001
Average egg weight (g)	53.85 ± 0.85	54.84 ± 1.59	0.208
feed/egg ratio	2.77 ± 0.09 ^a^	2.52 ± 0.12 ^b^	0.003
ADFI (g)	101.51 ± 1.38	101.73 ± 0.90	0.749

CON, control group; PE, plant extracts of *Flos lonicerae* and *Baikal skullcap* group. The data are expressed as the mean ± standard deviation (*n* = 6 per group). Different letters within a row denote a statistically significant difference (*p* < 0.05).

**Table 2:** Effect of PE increment on relative abundance of cecal microbiota species at genus level.

Items	CON	PE	*p*-Value
Bacteroides	20.09 ± 2.10 ^a^	14.37 ± 1.45 ^b^	0.004
Phascolarctobacterium	8.31 ± 4.54	8.90 ± 3.88	0.850
unclassified_f__Lachnospiraceae	4.52 ± 0.49	5.35 ± 0.58	0.072
Ruminococcus_torques_group	2.90 ± 1.26	3.86 ± 1.25	0.319
Prevotellaceae_UCG-001	4.36 ± 3.19	2.02 ± 0.91	0.207
Faecalibacterium	2.79 ± 1.58	2.40 ± 0.87	0.680
norank_f__norank_o__Clostridia_UCG-014	1.78 ± 0.71	2.61 ± 0.91	0.197
Desulfovibrio	2.12 ± 0.96	1.93 ± 1.23	0.817
Subdoligranulum	1.74 ± 0.70	1.68 ± 0.91	0.916
Megamonas	2.10 ± 1.59	1.31 ± 1.43	0.487
norank_f__Eubacterium_coprostanoligenes_group	1.24 ± 1.38	2.03 ± 1.40	0.454
Butyricicoccus	0.98 ± 0.26 ^b^	1.99 ± 0.56 ^a^	0.017
norank_f__Muribaculaceae	1.60 ± 0.48	1.09 ± 0.31	0.125
Lactobacillus	1.02 ± 0.48	1.08 ± 0.37	0.852
Christensenellaceae_R-7_group	0.77 ± 0.74	1.29 ± 0.62	0.320
Romboutsia	0.81 ± 0.81	0.73 ± 0.54	0.875
Alistipes	0.78 ± 0.41	0.62 ± 0.13	0.480
Colidextribacter	0.29 ± 0.24	0.66 ± 0.31	0.111
norank_f__Prevotellaceae	0.83 ± 0.78	0.09 ± 0.10	0.109

CON, control group; PE, plant extracts of *Flos lonicerae* and *Baikal skullcap* group. The data are expressed as the mean ± standard deviation (*n* = 4 per group). Different letters within a row denote a statistically significant difference (*p* < 0.05).

**Table 3:** Differential metabolites between the CON and PE groups.

Items	VIP	*p*-Value	FC	Trend
Leu-Enkephalin	1.51	<0.01	117.36	↑
Palmitoyl Serotonin	1.76	<0.01	48.33	↑
Repaglinide	1.74	<0.01	45.68	↑
2′-Hydroxy-4,4′,6′-trimethoxychalcone	1.71	<0.01	44.85	↑
Fenaminosulf	1.45	<0.01	35.89	↑
Lasalocid	1.72	<0.01	31.94	↑
2-Methoxyestradiol	1.73	<0.01	29.53	↑
Amsacrine	1.66	<0.01	26.08	↑
Vanillin	1.72	<0.01	24.75	↑
Nicotinic Acid Adenine Dinucleotide	1.58	<0.01	23.59	↑
Carboplatin	1.63	<0.01	22.55	↑
Enalapril	1.64	<0.01	21.84	↑
Bosentan	1.57	<0.01	21.12	↑
Rosuvastatin	1.56	<0.01	21.10	↑
Urobilin	1.71	<0.01	0.01	↓
Paxilline	1.78	<0.01	0.01	↓
Hexaconazole	1.57	<0.01	0.01	↓

CON, control group; PE, plant extracts of *Flos lonicerae* and *Baikal skullcap* group. The VIP value was obtained based on the OPLS-DA model. The divergences in metabolites between the control group and the placebo treatment group were evaluated by combining the *p*-value derived from the test and the fold-change (FC) of the metabolites. Screening of differential metabolites with the following criteria: FC ≤ 0.02 or FC > 20, *p* < 0.01 and VIP > 1. The upward pointing arrow denotes a significant upregulation of metabolites within the PE group in comparison to the CON group. The downward pointing arrow denotes that the metabolites were repressed in the PE group relative to the CON group.

## Data Availability

The data that support the findings of this study are available from the corresponding author upon reasonable request.

## Abbreviations

The following abbreviations are used in this manuscript:

PE	*Flos lonicerae* and *Baikal skullcap* extracts
CGA	Chlorogenic acid
CON	Control group
ADFI	Average daily feed intake
SWF	Small white follicles
LWF	Large white follicles
SYF	Small yellow follicles
LYF	Large white follicles
GSH-Px	Glutathione peroxidase
T-SOD	Superoxide dismutase
CAT	Catalase
T-AOC	Total antioxidant capacity
MDA	malondialdehyde
IgY	Immunoglobulins Y
IgA	Immunoglobulins A
IgM	Immunoglobulins M
ELISA	Enzyme-linked immunosorbent assay
IL-6	Interleukin-6
TNF-α	Tumor necrosis factor-alpha
IFN-γ	Interferon-γ
IL-1β	Interleukin-1β
E_2_	Estradiol
FSH	Follicle-stimulating hormone
LH	Luteinizing hormone
ASV	Amplicon Sequence Varian
PCoA	Principal Coordinates Analysis
LDA	Linear discriminant analysis
LEfSe	Linear discriminant analysis effect size
VIP	variable importance in projection
FC	Fold change
L-ENK	Leu-enkephalin
2ME	2-methoxyestradiol
NAD	Nicotinic acid adenine dinucleotide
NAM	Nicotinate and nicotinamide
ROS	Reactive oxygen species
SCFA	Short-chain fatty acid
HPOA	Hypothalamic-pituitary-ovarian axis

## Appendix A

**Table A1:** Composition and nutrient levels of the control diet (% dry matter).

Items	Content
Ingredients	
Corn	60.30
Soybean meal	24.70
Soybean oil	1.00
Limestone	9.50
Dicalcium phosphate	2.00
Sodium chloride	0.30
DL-Methionine	0.20
Premix ^1^	2.00
Total	100.00
Nutrient levels ^2^	
Metabolic energy (ME)/(MJ/kg)	10.88
Crude protein	15.70
Total phosphorus	0.66
Available phosphorus	0.48
Calcium	4.00
Methionine ^3^	0.42
Methionine + cysteine ^3^	0.72
Lysine ^3^	0.83

^1^ Provided per kg of diet: Vitamin A, 11,000 IU; Vitamin D3, 3000 IU; Vitamin E, 20 mg; Vitamin K3, 3 mg; Vitamin B1, 2.5 mg; Vitamin B2, 8.5 mg; Vitamin B6, 4 mg; Vitamin B12, 0.025 mg; nicotinic acid, 40 mg; D-pantothenate calcium, 14 mg; folic acid, 0.8 mg; D-biotin, 0.125 mg; choline chloride, 500 mg; copper, 8 mg; manganese, 100 mg; iron, 80 mg; zinc, 85 mg; iodine, 0.8 mg; selenium, 0.3 mg. ^2^ ME was a calculated value, while the others were measured values. ^3^ The amino acid values were expressed as total.
